# Emergence of a Novel Dengue Virus 3 (DENV-3) Genotype-I Coincident with Increased DENV-3 Cases in Yangon, Myanmar between 2017 and 2019

**DOI:** 10.3390/v13061152

**Published:** 2021-06-16

**Authors:** Aung Min Soe, Mya Myat Ngwe Tun, Takeshi Nabeshima, Theingi Win Myat, Moh Moh Htun, Htin Lin, Nang Sarm Hom, Shingo Inoue, Khine Mya Nwe, Lynn Pa Pa Aye, Mizuki Fukuta, Kyaw Zin Thant, Futoshi Hasebe, Kouichi Morita, Sujan Shresta, Hlaing Myat Thu, Meng Ling Moi

**Affiliations:** 1 Department of Virology, Institute of Tropical Medicine, Nagasaki University, Nagasaki 852-8523, Japan; dr.aungminnsoe@gmail.com (A.M.S.); myamyat@tm.nagasaki-u.ac.jp (M.M.N.T.); mtmikami@tm.nagasaki-u.ac.jp (T.N.); pampanga@nagasaki-u.ac.jp (S.I.); drkhinemyanwe@gmail.com (K.M.N.); mixgoldenretriever@gmail.com (M.F.); moritak@nagasaki-u.ac.jp (K.M.); 2 Graduate School of Biomedical Sciences, Nagasaki University, Nagasaki 852-8523, Japan; rainbow@nagasaki-u.ac.jp; 3 Program for Nurturing Global Leaders in Tropical and Emerging Communicable Diseases, Nagasaki University, Nagasaki 852-8501, Japan; 4 Department of Medical Research, Yangon 11191, Myanmar; drtheingiwinmyat@gmail.com (T.W.M.); mohmoh.htun@gmail.com (M.M.H.); drhtinlin@gmail.com (H.L.); sarmhom@gmail.com (N.S.H.); dr.lynnpapaaye@gmail.com (L.P.P.A.); drkz.thant@gmail.com (K.Z.T.); 5 Research Center for Infectious Disease Research in Asia and Africa, Institute of Tropical Medicine, Nagasaki University, Nagasaki 852-8523, Japan; 6 La Jolla Institute for Immunology, La Jolla, CA 92037, USA

**Keywords:** dengue outbreak, Myanmar, co-circulation, serotypes, genotype-1, DENV-3

## Abstract

Dengue fever, caused by the mosquito-borne dengue virus (DENV), has been endemic in Myanmar since 1970 and it has become a significant public health burden. It is crucial that circulating DENV strains are identified and monitored, and that their transmission efficiency and association with disease severity is understood. In this study, we analyzed DENV-1, DENV-2, DENV-3, and DENV-4 serotypes in 1235 serum samples collected in Myanmar between 2017 and 2019. Whole-genome sequencing of DENV-1–4 demonstrated that most DENV-1–4 strains had been circulating in Myanmar for several years. We also identified the emergence of DENV-3 genotype-I in 2017 samples, which persisted through 2018 and 2019. The emergence of the strain coincided with a period of increased DENV-3 cases and marked changes in the serotype dynamics. Nevertheless, we detected no significant differences between serum viral loads, disease severity, and infection status of individuals infected with different DENV serotypes during the 3-year study. Our results not only identify the spread of a new DENV-3 genotype into Yangon, Myanmar, but also support the importance of DENV evolution in changing the epidemic dynamics in endemic regions.

## 1. Introduction

Dengue virus (DENV) infection can be asymptomatic or cause an array of clinical symptoms that can include mild aches and pains, severe flu-like symptoms, and, less commonly, severe dengue composed of severe bleeding, organ impairment, plasma leakage/hypovolemic shock, and death [1,2,3,4]. In recent years, the incidence of DENV infection has increased in Myanmar, and the disease burden is now one of the largest among Southeast Asian countries.

DENV is a member of the *Flavivirus* genus of the *Flaviviridae* family of single-stranded positive sense RNA viruses [5] and exists as four closely related serotypes, DENV-1-4 [6,7]. Although other regions in Southeast Asia have reported dengue outbreaks since the 1950s, Myanmar recorded its first outbreak in the 1970s. Since then, the prevalence of DENV infection has increased, particularly in recent years [8]. All four DENV serotypes have been detected in Myanmar, with DENV-1 was being the most dominant serotypes, while DENV-4 has been detected at a lesser frequency, since 2000s [9,10]. The largest DENV-1 epidemic in Myanmar to date occurred in 2015, when 42,913 cases and 140 deaths were recorded [11]. Recent studies have suggested that dengue epidemics are being shaped by the emergence of new DENV strains [12,13]. Thus, there is an urgent need to document and track the serotypes and genotypes of DENV circulating in Myanmar, not only to gain a better understanding of the epidemiological factors that drive DENV outbreaks, but also to facilitate the implementation of effective DENV control measures, including vaccination programs. Phylogenetic analyses have demonstrated the regular extinction of existing strains and emergence of new strains, the latter of which tends to coincide with increased viral transmission [14]. In turn, transmission may be further amplified by the introduction of new strains from other regions or the appearance of strains with higher transmission fitness resulting from selective pressures [14]. To better understand which DENV serotypes and genotypes have been circulating in Yangon, Myanmar in recent years, we performed full-genome next-generation sequencing and phylogenetic and nucleotide variation analyses of DENV-1–4 isolates collected in Myanmar between 2017 and 2019. We demonstrate changes in DENV dynamics in this endemic region and identify a newly emerged DENV-3 genotype-I (GI) that co-circulated with DENV-3 genotype-III (GIII) during this time.

## 2. Materials and Methods

### 2.1. Patients and Sample Collection

The study protocol was approved by the Institutional Review Boards of the two participating hospitals, which are Yangon General Hospital (patients >12 years old) and Yangon Children Hospital (patients ≤12 years old). All patients or their legal guardians provided written informed consent. From 1235 individuals with suspected DENV infection, total of 1418 serum samples were collected (1235 acute serum samples and 183 convalescent serum samples) between June 2017 and December 2019. Suspected DENV infection was initially diagnosed based on clinical findings, and disease severity was classified according to the World Health Organization dengue diagnostic guideline (WHO, 2009). Initial DENV diagnostic testing was performed with SD BIOLINE Dengue NS1 Ag test kits (Standard Diagnostic Inc., Suwon, Korea). All serum samples were kept at −80 °C until analysis.

### 2.2. Serological Confirmation of DENV Infection Status

Serological confirmation of DENV infection and determination of infection status was performed using in-house anti DENV IgM-capture and anti-DENV IgG antibody enzyme-linked immunosorbent assays (ELISAs), and optical density (OD) values were read at 497 nm [15]. Sera were considered positive for IgM antibodies when the P/N ratios were ≥2. To distinguish the infection status (primary and secondary dengue virus infections) in-house anti-DENV IgG ELISA system [15] was used. The test highly correlates with the dengue hemagglutination inhibition (DEN HI) test [16].

### 2.3. Virus Isolation, RNA Extraction, and DENV Serotyping by RT-PCR

Cultured *Aedes albopictus* mosquito clone cells (C6/36) were resuspended in Eagle’s minimum essential medium supplemented with 2% fetal bovine serum (FBS) and 0.2 mM non-essential amino acid. The cells were seeded in Nunclon Delta (flat) tubes (Thermo Fisher, Waltham, MA, USA), mixed with 10 μL of patient serum and incubated at 28 °C for 7 days. The culture supernatants were then harvested, transferred to fresh cultures of C6/36 cells, and the tubes were incubated for an additional 7 days [17]. After a total of 14 days, the cell culture supernatant was collected, and viral RNA was extracted and analyzed by reverse transcription-polymerase chain reaction (RT-PCR). Briefly, RNA was extracted using QIAamp RNA mini kits (Qiagen GmbH, Hilden, Germany) and amplified using Takara One-Step RT-PCR kits (Takara Bio Inc., Shiga, Japan) using consensus and DENV serotype-specific primers [18,19].

### 2.4. Whole-Genome Sequencing

First strand cDNA was synthesized from RNA that was isolated from the culture supernatants. Next, Superscript III and random primers (Thermo Fisher, Waltham, MA, USA) was used to generate the first strand cDNA, and the second strand cDNA was synthesized using NEBNext Second Strand Synthesis kits (New England Biolabs, Ipswich, MA, USA). DNA libraries were prepared using a Nextera XT DNA library prep kit (Illumina, San Diego, CA, USA) with aliquots of 1 ng of DNA for tagmentation. Samples were quantified using a Qubit High Sensitivity dsDNA kit (Thermo Fisher, Waltham, MA, USA). Paired-end sequencing was performed using an Illumina MiSeq system with MiSeq sequencing reagent kits (v2, 2 × 250 bp; Illumina, San Diego, CA, USA).

### 2.5. Phylogenetic Analysis and Amino Acid Variant Analysis

The FASTX-Toolkit v0.0.14 was used for analysis. Adaptor sequences were trimmed, and quality was checked with FastQC v0.11.8 [20]. Trinity v2.8.4 was used for de novo assembly [21] and Blastn v2.7.1 [22] was used for contig assembly. Sequence identity was modified with SeqKit v10.0.1 [23] and fastq data was mapped with bwa v0.7.17 [24]. Amino acid variants were detected using Lofreq [25] and Varscan v2.4.3 [26], and the results were counter-checked with Pilon [27], V-phaser-2 [28], SNVer, and freebayes [29]. SAMtools v1.9 was then used to generate the consensus sequence [30]. Pre-processing of the data was performed using GATK v3.8.1 and Picard v2.20 [30]. Global sequences were obtained from the International Nucleotide Sequence Database Consortium and annotation was performed using SeqKit. Sequences were aligned using Mafft v7.407 [31]. The maximum likelihood phylogenetic tree was created with PhyML v3.2.0 [32] with 1000-replication bootstrap values, and substitution model (jModelTest v2.1.10) [33].

### 2.6. Sanger Sequencing

The E-gene region was also sequenced and confirmed by the Sanger method [10] using a Bigdye Dideoxy Terminator Sequencing kit v3.1 (Applied Biosystems, Foster, CA, USA), according to the manufacturer’s instructions. Sequences were purified using Agencourt CleanSEQ kit (Agencourt Bioscience, Beverly, MA, USA) and analyzed using a 3730 DNA Analyzer (Applied Biosystems, Foster, CA, USA) [34].

### 2.7. Quantification of DENV in Serum Samples

Viremia levels in serum samples were quantified using two assays: an infectious virus plaque assay with the FcγR-expressing BHK cell line [35] and a quantitative (q) RT-PCR assay.

For the plaque assay, BHK cells were resuspended in Eagle’s Minimum Essential Medium (Sigma-Aldrich, Gillingham, UK) supplemented with 10% FCS and 0.5 mg/mL neomycin (G418) (PAA Laboratories GmbH, Pasching, Austria) and grown to confluence. The serum samples were serially diluted 10-fold (1:100–1:10,000) with EMEM, an aliquot of 200 μL of each dilution was added to the confluent BHK cells, and the plates were incubated at 37 °C for 1 h. The cells were then overlaid with 1.5 mL of maintenance medium and the plates were incubated at 37 °C with 5% CO_2_ for 5 days. The cells were then fixed with 4% paraformaldehyde, stained with 0.25% crystal violet, and visualized under an inverted microscope. Plaques were counted and viral levels are expressed as plaque forming units (PFU) per mL of serum.

For qRT-PCR analysis, 5 μL of RNA (extracted from the serum samples directly) was amplified the envelope gene by TaqMan real time RT-PCR system (Life Technologies, Carlsbad, CA, USA) [30,36]. Virus genome levels were expressed as log10 genome copies per mL of serum.

### 2.8. Statistical Analyses

Data were analyzed using Prism v9.0.2 software (GraphPad, San Diego, CA, USA) and compared using one way analysis of variance (ANOVA) and Student’s *t*-test. A *p* value of <0.05 was considered statistically significant.

## 3. Results

### 3.1. Clinicopathological Characteristics and Serostatus of Patients

During the study period, 1235 acute serum samples were obtained; of these, 912 (73.8%) samples were NS1 antigen positive (SD BIOLINE Dengue NS1 Ag test kits) between June 2017 and December 2019. To confirm positivity and assess infection status, these 912 samples were subjected to ELISA tests. In-house dengue IgM capture ELISA tests produced a positive result for 776 (85.1%) of the 912 total cases. In-house dengue IgG indirect ELISA tests was used to identify 264 (28.9%) cases as primary infection and, 648 (71.1%) as secondary infection (Figure S1). During the study period, the number of hospitalized patients per year was highest in 2018, as indicated by analysis of the weekly hospitalization rate for 2017–2019 (Figure S2).

### 3.2. Distribution of Circulating DENV Serotypes

Among the 912 NS1-positive samples, a total of 212 isolates was obtained by using the C6/36 clone cells, and there was no mixed infection (Table 1). The majority of isolates collected in 2017 were DENV-4 (*n* = 20) and DENV-3 (*n* = 18) serotypes, whereas in both 2018 and 2019, DENV-3 (*n* = 55 and 25, respectively) and DENV-1 (*n* = 30 and 20, respectively) were the predominant serotypes. Over the entire 3-year period, the most prevalent serotype was DENV-3 (*n* = 98, 46.2%), followed by DENV-1 (*n* = 64, 30.1%), and DENV-4 (*n* = 42, 19.8%). Notably, DENV-2 was detected in only 8 of the 212 isolates during the study period.

The majority of the patients that was positive by virus isolation were children ≤12 years of age (160 out of 212, 75.5%) (Table 2). The mean age was 9.7 (±6.3) years and consisted of 120 (56.7%) male and 92 (43.3%) female participants. According to the WHO classification for dengue symptoms, 74 (34.9%), 119 (56.1%), and 19 (9%) patients were classified as having dengue without warning signs (DWoWS), dengue with warning signs (DWWS) and severe dengue (SD), respectively.

### 3.3. Whole-Genome Sequencing and Phylogenetic Analysis

**Figure 1:**
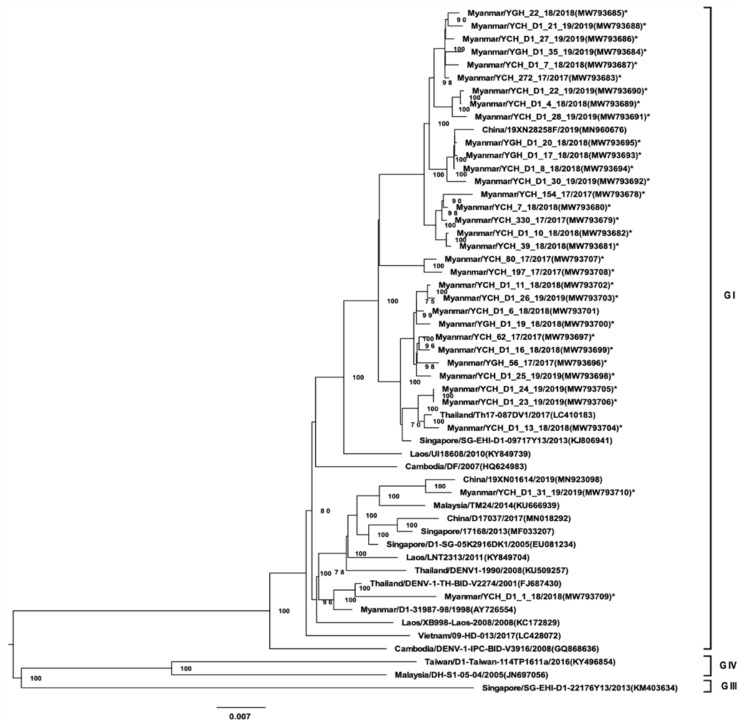
Phylogenetic tree of DENV-1 based on whole-genome sequencing. The maximum likelihood tree was generated using a general time reversible GTR + I + G model with 1000 bootstrap replicates. Bootstrap values of >70 were indicated in the nodes. The tree shows global DENV strains, and the 33 DENV-1 isolates from this study (indicated by asterisks). These and representative strains of each genotype are named by country of origin, strain name, year of isolation, and accession number (GenBank).

**Figure 2:**
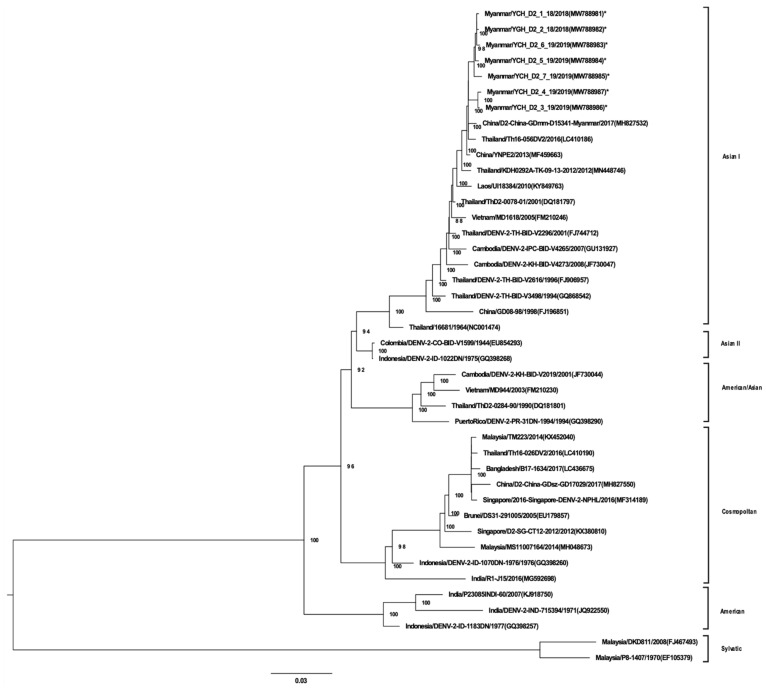
Phylogenetic tree of DENV-2 based on whole-genome sequencing. The maximum likelihood tree was generated using a general time reversible (GTR + I + G) model with 1000 bootstrap replicates. Bootstrap values of >70 were indicated in the nodes. The tree shows global DENV strains, and the 7 DENV-2 isolates from this study are indicated by asterisks. Representative strains of each genotype are named by country of origin, strain name, year of isolation, and accession number (GenBank).

**Figure 3:**
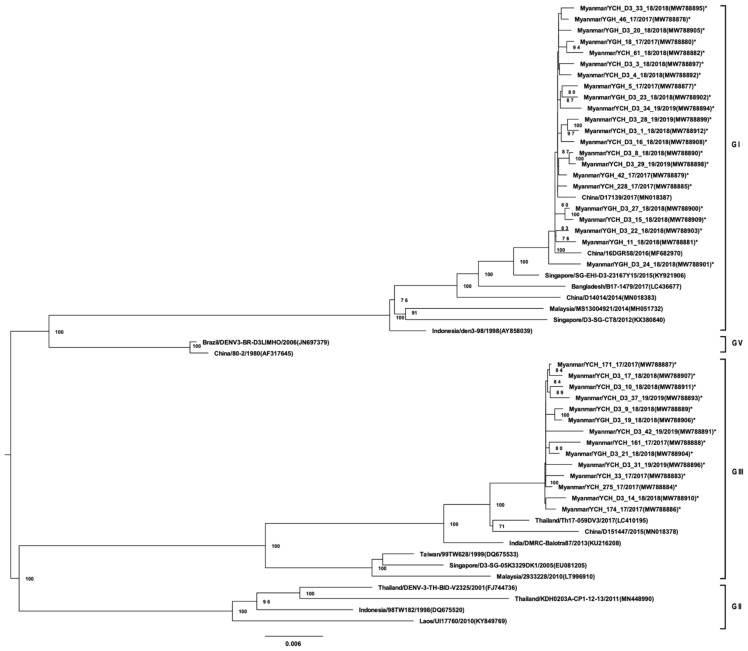
Phylogenetic tree of DENV-3 based on whole-genome sequencing. The maximum likelihood tree was generated using a general time reversible (GTR + I + G) model with 1000 bootstrap replicates. Bootstrap values of >70 were indicated in the nodes. The tree shows global DENV strains, and the 36 DENV-3 isolates from this study are indicated by asterisks. Representative strains of each genotype were named by country of origin, strain name, year of isolation, and accession number (GenBank).

**Figure 4:**
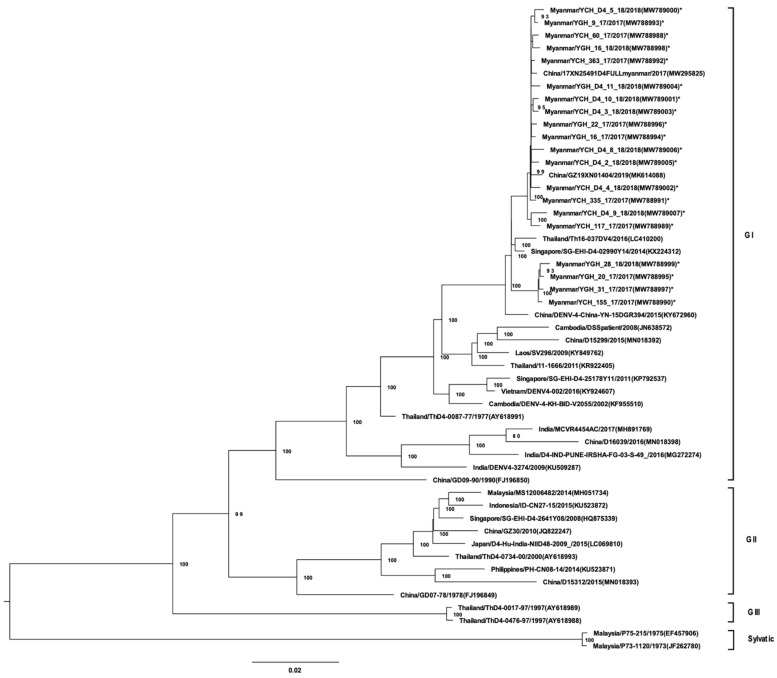
Phylogenetic tree of DENV-4 based on whole-genome sequencing. The maximum likelihood tree was generated using a general time reversible (GTR + I + G) model with 1000 bootstrap replicates. Bootstrap values of >70 were indicated in the nodes. The tree shows global DENV strains, and the 20 DENV-4 isolates from this study are indicated by asterisks. Representative strains of each genotype were named by country of origin, strain name, year of isolation, and accession number (GenBank).

A total of 96 virus isolates obtained from a second cell culture passage were analyzed by NGS and amino acid variants. The isolates was selected based on positive isolation during the second passage by random sampling. All DENV-1 isolates (33 strains) belonged to GI and, possessed high homology to DENV-1 strains from regions neighboring Myanmar (Figure 1). DENV-1 GI was associated with two distinct lineages in Myanmar. The 7 DENV-2 isolates belonged to Asian GI (Figure 2). Of the 36 DENV-3 isolates, 22 were of GI and were phylogenetically closely related to strains found in China, Singapore, Bangladesh, and Malaysia (Figure 3). Notably, DENV-3 GI was not detected in Myanmar prior to 2017. Phylogenetic analyses suggested that the novel DENV-3 GI was present in Myanmar by June 2017 and has remained in circulation since then. Although transmission of DENV-4 increased during 2017–2019, the circulating genotype (GI) remained unchanged from that detected in previous years (Figure 4).

**Figure 5:**
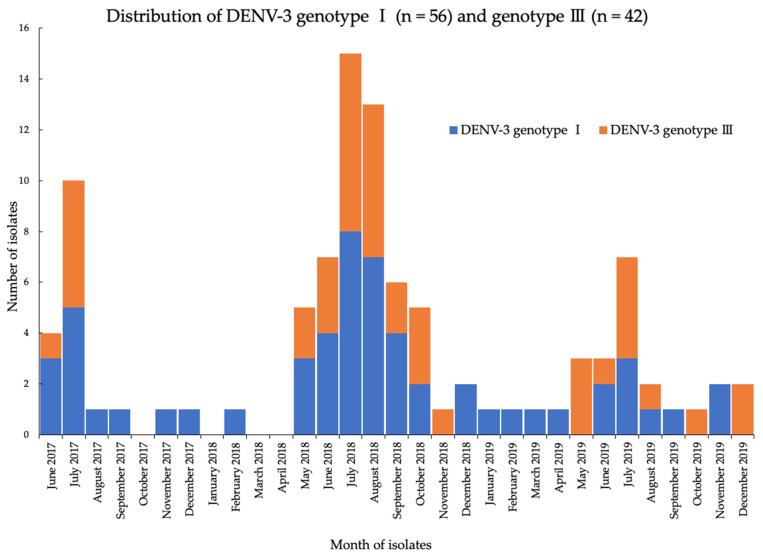
Distribution patterns of DENV-3 genotypes-I (blue) and -III (orange) co-circulating in Yangon, Myanmar during 2017–2019.

To elucidate the distribution patterns of the two genotypes of DENV-3 across the study period, partial E gene sequencing analyses of DENV-3 strains were performed by using Sanger sequencing (62 strains) and NGS (36 strains) (Figure 5). Overall, the results indicate that the DENV-3 genotype 1 had emerged in the country by June 2017, and it was maintained throughout the study period. In 2018 and 2019, nearly equal distribution of genotypes-1 and -3 was observed.

### 3.4. DENV Amino Acid Variants

**Figure 6:**
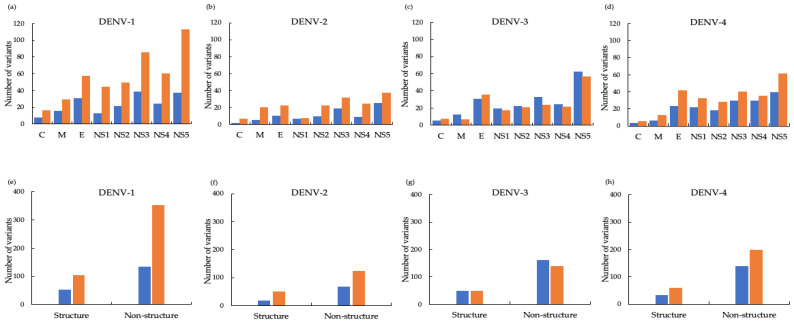
Amino acid variants of DENV-1–4 co-circulating in Yangon, Myanmar between 2017 and 2019. (**a**–**d**) Number of variants harboring synonymous (blue) and non-synonymous (orange) amino acid changes in the capsid (**C**), membrane-associated (**M**), envelope (**E**), and non-structural (NS1–5) proteins of (**a**) DENV-1, (**b**) DENV-2, (**c**) DENV-3, and (**d**) DENV-4. (**e**–**h**) Total number of variants with amino acid changes in structural (**C**,**M**,**E**) and non-structural (NS1–5) proteins. In total, 452 and 184, 169 and 82, 185 and 206, and 254 and 168 non-synonymous (orange) and synonymous (blue) variants, respectively, were identified for DENV-1, DENV-2, DENV-3, and DENV-4, respectively. For DENV-1, the number of NSY and SY variants in non-structural proteins was 350 and 132, respectively, compared with 102 and 52, respectively, in the structural proteins. The three most frequently mutated proteins for DENV-1 were NS5 (NSY 112, SY 37), followed by NS3 (NSY 85, SY 38), and E (NSY 57, SY30). Similarly, more DENV-2 variants harbored NSY than SY changes and more changes affected non-structural proteins (NSY 121 and SY 66, respectively) than structural proteins (NSY 48 and SY 16, respectively). The most frequently mutated DENV-2 proteins were NS5 (NSY 37, SY 25), NS3 (NSY 31, SY 18), and E (NSY 22, SY 10). For DENV-3, the total number of NSY and SY variants in non-structural proteins was 137 and 159, respectively, and in structural proteins was 48 and 47, respectively. The most frequently mutated DENV-3 proteins were NS5 (NSY 56, SY 62), E (NSY 35, SY 30), and NS3 (NSY 23, SY 32). Finally, for DENV-4, the total number of NSY and SY variants in non-structural proteins was 196 and 136, respectively, and in structural proteins was 58 and 32, respectively. The most frequently mutated were NS5 (NSY 61, SY 39), E (NSY 40, SY 23), and NS3 (NSY 40, SY 29).

We also analyzed amino acid variants in the DENV-1–4 isolates collected (Figure 6). Non-synonymous (NSY) and synonymous (SY) amino acid variants were identified by comparison to the reference strains DENV-1 AY726554, DENV-2 MF459663, DENV-3 KY921906, and DENV-4 KY672960.

Overall, amino acid variants of each serotype were predominantly due to NSY changes in all proteins, with the exception of DENV-3, which consisted of more SY variants than NSY variants (Figure 6). The majority of variants for all four DENV serotypes demonstrated NSY alterations in the NS5 protein, followed by NSY changes in the NS3 or E proteins.

### 3.5. Associations between Viremia, Disease Severity, and Infection Status

**Figure 7:**
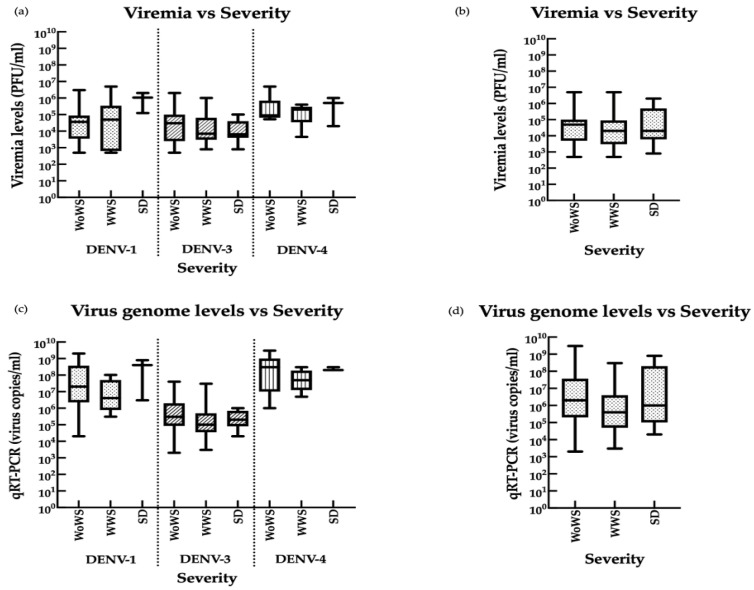
Comparison of viremia and disease severity in patients infected with DENV-1, DENV-3, and DENV-4. (**a**–**d**) Serum DENV-1 (*n* = 31), DENV-3 (*n* = 76), and DENV-4 (*n* = 15) levels determined by (**a**,**b**) plaque assay and (**c**,**d**) qRT-PCR. Data are presented for each serotype individually (**a**,**c**) or in combination (**b**,**d**). WoWS, without warning signs; WWS, with warning signs; SD, severe dengue (WHO classification). Boxplots show the median values (horizontal line in the box), 25–75% interquartile range (lower–upper limits of the box).

To determine whether DENV-1, -3, and -4 infection loads correlated with disease severity in viremic patients, we quantified infectious virus by plaque assays and viral genomic copies by qRT-PCR (Figure 7). DENV-2 was not analyzed due to the small sample number (*n* = 8). Using the WHO classification system, we found no significant differences in viral levels between patients classified as having DWoWS, DWWS, or SD (Figure 7), although patients with SD showed a trend towards higher DENV-1 and DENV-4 burden measured by both assays. Similarly, although mean viral levels demonstrated a tendency to be higher in patients with primary infection than secondary infection, the differences were not statistically significant (Figure S3).

## 4. Discussion

Records of DENV serotypes and transmission in Myanmar before 2000 showed no distinct patterns; however, DENV-1 emerged as the dominant serotype in 2001 [9]. Between 2000 and 2017, DENV-1 was associated with several large disease outbreaks [9,10,11]. Although Myanmar and the surrounding region has been categorized as DENV endemic, relatively little is known about DENV outbreaks associated with serotypes other than DENV-1. Similarly, few studies have evaluated the evolutionary trajectory of DENV or the associations between serotype prevalence and clinical presentation. To address this gap, we performed a comprehensive analysis of the DENV serotypes and genotypes circulating in Myanmar between 2017 and 2019 and analyzed the association between the circulating DENV serotypes and clinical outcomes.

Phylogenetic analyses of DENV-1 performed since 2000, 2013, and 2015 have identified several major lineage turnovers is associated with DENV outbreaks and serotype dominance. In this study, we found that DENV-1, DENV-3, and DENV-4 serotypes were represented at approximately the same rates in 2017, despite the dominance of DENV-1 in earlier years. By 2018, the transmission dynamics in Myanmar had changed such that DENV-3 became the major circulating serotype in 2018 and 2019, followed by DENV-1. Thus, it is possible that serotype displacement and subsequent genotype shifting may be responsible for the sharp increase in DENV cases between 2017 and 2019, and may have contributed to the higher mortality rates (nearly 200 deaths) seen in 2017.

In this study, we did not detect any changes in the genotypes of DENV-1, DENV-2, or DENV-4 circulating in Yangon, Myanmar, between 2017 and 2019. However, we identified a novel DENV-3 GI that emerged early in 2017 and persisted through the 3-year study period. This emergent DENV-3 GI was identified as the predominant genotype during several major dengue outbreaks in Southeast Asian countries, including Thailand. DENV-3 GI was first detected in Thailand in 1988 [37], after which it increased, receded, and then reappeared during the 2015–2016 dengue season [38]. The strains identified here as circulating in Myanmar have high sequence homology with strains with high transmission activity circulating in neighboring countries (Indonesia, Malaysia, and Singapore) [39,40,41,42]. One of these countries is the most likely point of entry of the emergent strain into Myanmar, although the precise origin is unknown. The capital of Myanmar, Yangon, has a high population density and heavy human traffic due to socioeconomic factors. While DENV transmission in large urban areas has been suggested to be focal points for further spread to other regions, further studies are needed to determine whether the DENV transmission patterns in Yangon reflect those of other localities. While DENV-3 GI and GIII were co-circulating in Yangon, Myanmar, in 2017–2019, our results suggest that the emergent GI strain may have higher transmission activity, consistent with the increased infection rate in recent years.

Our analysis of DENV-1–4 amino acid variants revealed that NS5 was the most frequently mutated gene, followed by NS3 and E. The NS5 region encompasses the polymerase region that is highly conserved between serotypes and plays a key role in maintaining viral fitness and replication capacity [43]. Most of the variants of DENV-1, DENV-2, and DENV-4 were non-synonymous, while DENV-3 variants were predominantly due to synonymous changes in the NS5, NS3, and E proteins. This finding suggests that genotype expansion may have resulted from a combination of introduction of a new DENV-3 strain and its ongoing evolution at the local level. One factor proposed to be involved in driving of genotype/serotype turnover is genetic bottlenecks or positive selective pressures, which could confer an advantage for enhanced transmission that in turn leads to larger outbreaks.

Two hypotheses have been proposed to explain the marked differences in disease severity that can result from DENV infection: one is that viral mutation and evolution drive increased virulence [12] and the second is that pre-existing anti-DENV immunity may increase the severity of secondary infection through the process of antibody-enhancement of infection [13]. Although we found increases and decreases in the prevalence of DENV serotypes over the study period, we detected no significant differences between viremia, clinical severity and type of infection in patients infected with different serotypes. This result suggests that higher number of cases does not correlate with viral burden or clinical presentation; thus, further studies are warranted to determine whether an association exists between virus pathogenicity and transmission efficiency. Moreover, the observation that an increase in DENV-3 infections coincided with the emergence of a DENV-3 GI in Yangon, Myanmar suggests that the new strain may be more efficiently transmitted than the previously circulating strains. Further studies on viral dynamics and herd immunity will be important to understand the transmission dynamics of emergent strains in this DENV endemic country.

## 5. Conclusions

DENV infection is a major public health problem in Myanmar. We sequenced DENV serotypes circulating in Myanmar between 2017 and 2019 and compared them with historical data for DENV isolates spanning the preceding two decades. We identified a newly emerged DENV-3 GI that was introduced into Myanmar in 2017 and has persisted since then. Although a local DENV-3 GIII strain was also circulating in 2017–2019, the emergence of the DENV-3 GI coincided with a marked increase in DENV-3 transmission activity, suggesting that it may be the more pathological strain. Overall, our results suggest an important role for DENV genotype evolution in transmission dynamics in this endemic region, and additionally underscore the crucial need to monitor viral dynamics on an ongoing basis.

## Figures and Tables

**Table 1:** Distribution of DENV serotypes circulating in Yangon, Myanmar 2017–2019.

	Number of Isolates Per Year			
Serotype	2017	2018	2019	Total
DENV-1	14	30	20	64
DENV-2	0	3	5	8
DENV-3	18	55	25	98
DENV-4	20	18	4	42
	52	106	54	212

**Table 2:** Clinicopathological characteristics and laboratory findings of patients in the study cohort ^1^.

	Patients Positive for Each DENV Serotype (N, %)					
Patient Characteristic	DENV-1 (*n* = 64, 30.1%)	DENV-2 (*n* = 8, 3.8%)	DENV-3 (*n* = 98, 46.2%)	DENV-4 (*n* = 42, 19.8%)	Total (*n* = 212, 100%)	*p* Value
**Age (years)**	8.8 (±5.1) ^2^	8.0 (±4.6)	9.6 (±5.4)	11.7 (±5.4)	9.7 (±6.3)	
**Age group (years)**						
≤12	52 (32.5%)	7 (4.4%)	75 (46.8%)	26 (16.3%)	160 (100%)	0.111
>12	12 (23%)	1 (2%)	23 (44.2%)	16 (30.8)	52 (100%)	
**Gender**						
Male	30 (46.8%)	6 (75%)	64 (65.3%)	20 (47.6%)	120 (56.7%)	0.047
Female	34 (53.2%)	2 (25%)	34 (34.7%)	22 (52.4%)	92 (43.3%)	
**Signs and Symptoms**						
Rash	3	0	8	3	14	0.717
Hess test	62	7	94	38	201	0.038
Coffee ground vomiting	4	1	13	1	19	0.161
Muscle pain	6	1	19	12	38	0.081
Joint pain	6	1	17	10	34	0.241
Abdominal pain	21	2	31	15	69	0.932
Hepatomegaly	41	5	39	20	105	0.027
Splenomegaly	0	0	1	1	2	0.632
Drowsiness	17	2	31	8	58	0.493
Thrombocytopenia. (<150,000/μL platelets)	26	2	32	20	80	0.308
Epistaxis	11	2	19	5	37	0.689
Melena	0	0	7	1	8	0.106
**Symptoms ^3^**						
Without warning signs	27 (42.2%)	2 (25%)	27 (27.5%)	18 (42.8%)	74 (34.9%)	
With warning signs	32 (50%)	6 (75%)	62 (63.3%)	19 (45.2%)	119 (56.1%)	0.284
Severe dengue	5 (7.8%)	0	9 (9.2%)	5 (12%)	19 (9%)	
**Anti-DENV IgM ^4^**						
Negative	24 (37.5%)	3 (37.5%)	43 (43.8%)	12 (28.6%)	82 (38.7%)	0.397
Positive	40 (62.5%)	5 (62.5%)	55 (56.2%)	30 (71.4%)	130 (61.3%)	
**Type of infection ^5^**						
Primary	31 (48.4%)	1 (12.5%)	50 (51%)	5 (11.9%)	87 (41%)	<0.001
Secondary	33 (51.6%)	7 (87.5%)	48 (49%)	37 (88.1%)	125 (59%)	

^1^ Patients was confirmed positive by virus isolation, ^2^ Mean ± standard deviation, ^3^ WHO classification system, ^4^ as determined by capture ELISA, ^5^ as determined by in-house dengue IgG indirect ELISA, underline indicates statistically significant values (*p* value < 0.05).

## Data Availability

Dengue virus sequence data obtained from the samples in this study has been deposited in National Center for Biotechnology Information (NCBI) database (accessions number of each strain is as indicated in the virus strain name in Figure 1, Figure 2, Figure 3 and Figure 4). The datasets generated during and/or analyzed during the current study are available from the corresponding authors on reasonable request.

## Supplementary Materials

The following materials are available online at https://www.mdpi.com/article/10.3390/v13061152/s1, Figure S1: Flow diagram of the methods used for this study, Figure S2: Number of admitted cases per week per year of NS1 positive samples {2017 (*n* = 282), 2018 (*n* = 440) and 2019 (*n* = 190)} during the study period, Figure S3. Comparison of viremia and infection status in patients infected with DENV-1, DENV-3, and DENV-4.
