# Supplementary material for: Emergence of a Novel Dengue Virus 3 (DENV-3) Genotype-I Coincident with Increased DENV-3 Cases in Yangon, Myanmar between 2017 and 2019
Source: Viruses. 2021 Jun 16;13(6):1152. doi: 10.3390/v13061152 (PMC8235066; doi:10.3390/v13061152)

Supplementary Figure S1. Flow diagram of the methodology used in the study.

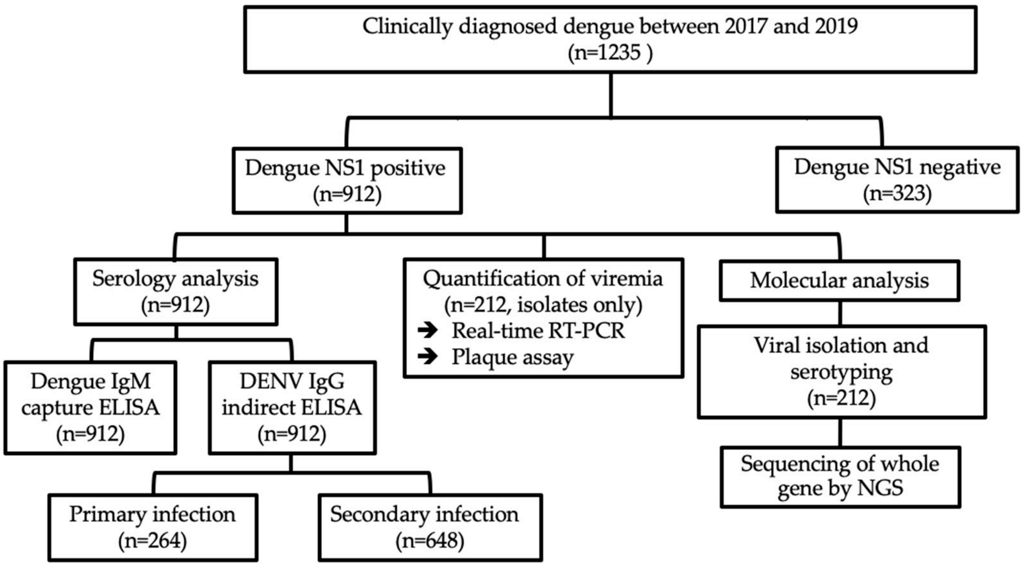

Supplementary Figure S2. Number of admitted cases per week per year of NS1 positive samples {2017 (n=282), 2018 (n=440) and 2019 (n=190)} during the study period.

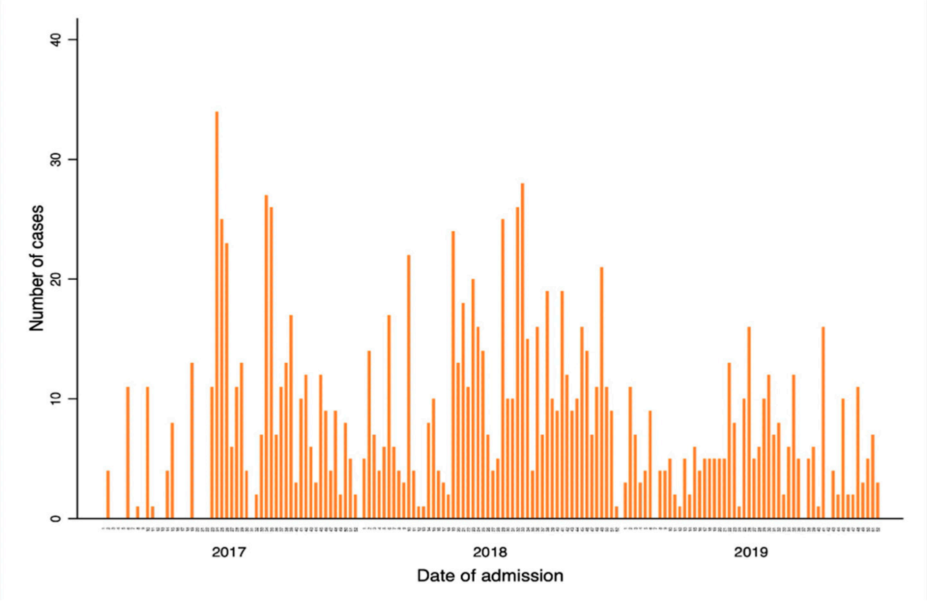

Supplementary Figure S3. Comparison of viremia and infection status in patients infected with DENV-1, DENV-3, and DENV-4. (a) Serum DENV-1 (n=31), DENV-3 (n=76), and DENV-4 (n=15) levels determined by (a, b) plaque assay and (c, d) qRT-PCR in patients with serologically defined primary and secondary infection. Data are presented for each serotype individually (a, c) or in combination (b, d). Horizontal bars represent the mean values and symbols represent individual samples. DENV-2 was not analyzed due to the small sample number (n=8).

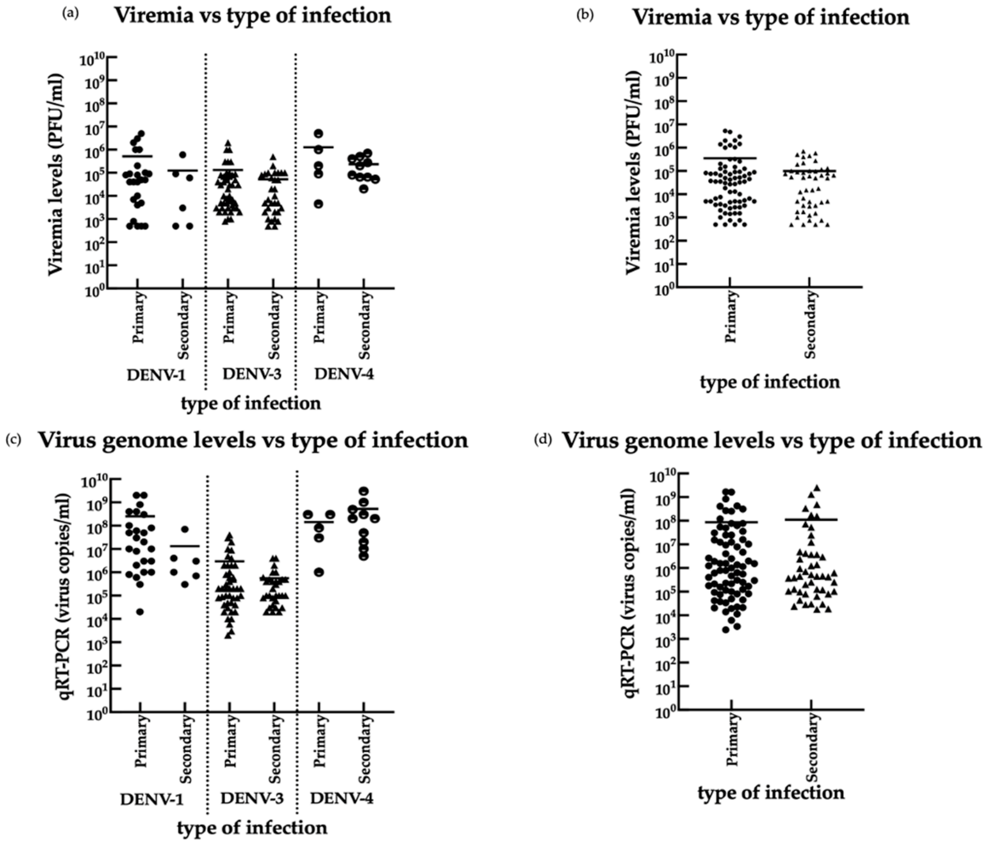

Supplement: Supplementary file 1 [file viruses-13-01152-s001.zip › viruses-1224307-supplementary.pdf]
